# Effect of Fiber Shape on the Tribological, Mechanical, and Morphological Behaviors of Sisal Fiber-Reinforced Resin-Based Friction Materials: Helical, Undulated, and Straight Shapes

**DOI:** 10.3390/ma14185410

**Published:** 2021-09-18

**Authors:** Siyang Wu, Jiale Zhao, Mingzhuo Guo, Jian Zhuang, Qian Wu

**Affiliations:** 1 Key Laboratory of Bionic Engineering, Ministry of Education, Jilin University, Changchun 130022, China; siyangwu@outlook.com (S.W.); zhuangjian_2001@163.com (J.Z.); 2 School of Biological and Agricultural Engineering, Jilin University, Changchun 130022, China; wuqian18@mails.jlu.edu.cn

**Keywords:** friction material, natural fiber, fiber shape, tribological behavior, morphological characterization

## Abstract

In this paper, we aim to evaluate the tribological, mechanical, and morphological performance of resin-based friction composites reinforced by sisal fibers with different shapes, namely helical, undulated, and straight shapes. The experimental results show that the shape of the sisal fibers exerts a significant effect on the impact property of the composite materials but no obvious influence on the density and hardness. The friction composite containing the helical-shaped sisal fibers exhibits the best overall tribological behaviors, with a relatively low fade (9.26%), high recovery (98.65%), and good wear resistance (2.061 × 10^−7^ cm^3^∙N^−1^∙m^−1^) compared with the other two composites containing undulated-shaped fibers and straight-shaped fibers. The impact fracture surfaces and worn surfaces of the composite materials were inspected by scanning electron microscopy, and we demonstrate that adding helical-shaped sisal fibers into the polymer composites provides an enhanced fiber–matrix interface adhesion condition and reduces the extent of fiber debonding and pullout, effectively facilitating the presence of more secondary plateaus on the friction surface, which are responsible for the enhanced tribological and mechanical properties. The outcome of this study reveals that sisal fibers with a helical shape could be a promising candidate as a reinforcement material for resin-based brake friction composite applications.

## 1. Introduction

Friction materials, as important parts of the brake system, are extensively used in the automotive, railway, air, and other similar transport fields [1,2]. These friction materials must fulfil certain performance requirements, including an adequate and stable friction property, low wear, low fade, desirable recovery behavior, less brake noise and vibration, a reasonable cost, easy machinability, and environmental friendliness [3,4,5]. As the above performance requirements cannot be satisfied by a single component, friction materials are usually manufactured as multi-component polymer composites, which contain at least 10 raw elements. According to their diverse functions in the friction materials, these raw elements are essentially divided into the following categories: reinforcement fibers, phenolic binders, friction modifiers (abrasives and lubricants), and particulate fillers (functional and inert) [6,7].

In friction polymer composites, reinforcement fibers are generally considered to be indispensable and significant components to maintain the tribological, mechanical, and thermal behaviors of the composites [8,9,10]. In the early stages, asbestos fibers were commonly used in the formulation of friction materials owing to their availability and good durability as well as a good thermal resilience [2]. The use of asbestos fibers was stopped due to their carcinogenic nature, which caused extremely harmful effects on the health of humans [11]. Since then, massive efforts have been made by scientists and academics to create effective substitutes for asbestos fibers. Metallic fibers (such as copper fiber and steel fiber) and synthetic fibers (such as aramid fiber and glass fiber) and their combinations were gradually applied in the friction material manufacturing industries [12,13,14]. However, these metallic and synthetic fibers as non-biodegradable materials adversely affect the water and air environment during their use and disposal. Recent trends have demonstrated a need for environmental sustainability and natural fibers are gaining significance as reinforcement components in friction composite systems.

Natural fibers show several favorable properties over traditional synthetic fibers, such as an eco-friendly nature, abundant availability, a reduced cost, a lower density, comparable mechanical performance, and less energy consumption during processing, as well as an easier manufacturing process for the polymer composites [15,16,17]. Natural fibers such as bamboo, corn stalk, flax, rattan, hemp, sisal, kenaf, and banana [11,18,19,20] have been used as reinforcing materials for friction composite formulations. Their tribo-mechanical effectiveness has also been investigated by various researchers over the past decade. Surya Rajan et al. [21,22] added *Prosopis juliflora* bark fibers (PJBFs) to reinforce epoxy resin-based friction materials and proved that the PJBFs significantly enhanced the thermomechanical and tribological behaviors of the polymer composites, which made them useful for braking applications. Liu et al. [23] fabricated an eco-friendly friction composite using alkali-treated abaca fibers and demonstrated that the inclusion of abaca fibers reasonably improved the frictional stability even at an elevated temperature, and also increased the impact strength and wear resistance behaviors of the polymer composites. Matějka et al. [24] assessed the friction and wear performance of non-asbestos organic friction polymeric composites containing jute fibers and provided evidence that jute fibers were promising reinforcement components for tribological applications. 

In previous investigations, researchers recognized that the fiber–matrix interface adhesion condition is a significant issue in the use of natural fibers as reinforcements in friction composite systems. Improper interface bonding easily results in inefficient stress transfer between the composite matrix and reinforcement fibers, and even causes fiber pullout failure during use, which reduces the overall tribological and mechanical behaviors of the polymer composites [25,26,27,28]. To overcome the above limitations and enhance the reinforcing efficiency of natural fibers, many researchers have proposed various surface treatments for natural fibers, such as alkalization, benzoylation, silanization, acrylation, stearic acid treatments, and potassium permanganate treatments, prior to polymer composite fabrication [29,30]. To date, plenty of published research has reported the effects of chemically treated fibers on the tribological and mechanical behaviors of friction composites. However, minimal research has been undertaken concerning the influence of the structural design of natural fibers on the properties of polymer composites. The fiber shape also has a certain effect on the fiber–matrix interface bonding, which further affects the overall performance of the fiber-reinforced friction materials.

Sisal fibers, belonging to the agave family, are one of the most widely used reinforcing components in composite materials. They demonstrate a relatively high specific strength, long fibers, and ease of processing compared with other natural fibers [31]. In the published literature [32], sisal fibers have been proven to be a potential reinforcement for brake friction materials. Therefore, the objectives of the present study were to develop resin-based polymer composites reinforced by sisal fibers with different shapes, including helical, undulated, and straight shapes, and to explore the influence of the fiber shape on the tribological and mechanical behaviors of the developed composite materials. The friction surfaces and fracture surfaces after tribological and mechanical testing were studied and analyzed using scanning electron microscopy (SEM) to reveal the corresponding underlying mechanisms.

## 2. Materials and Methods

### 2.1. Preparation of the Fibers

**Figure 1:**
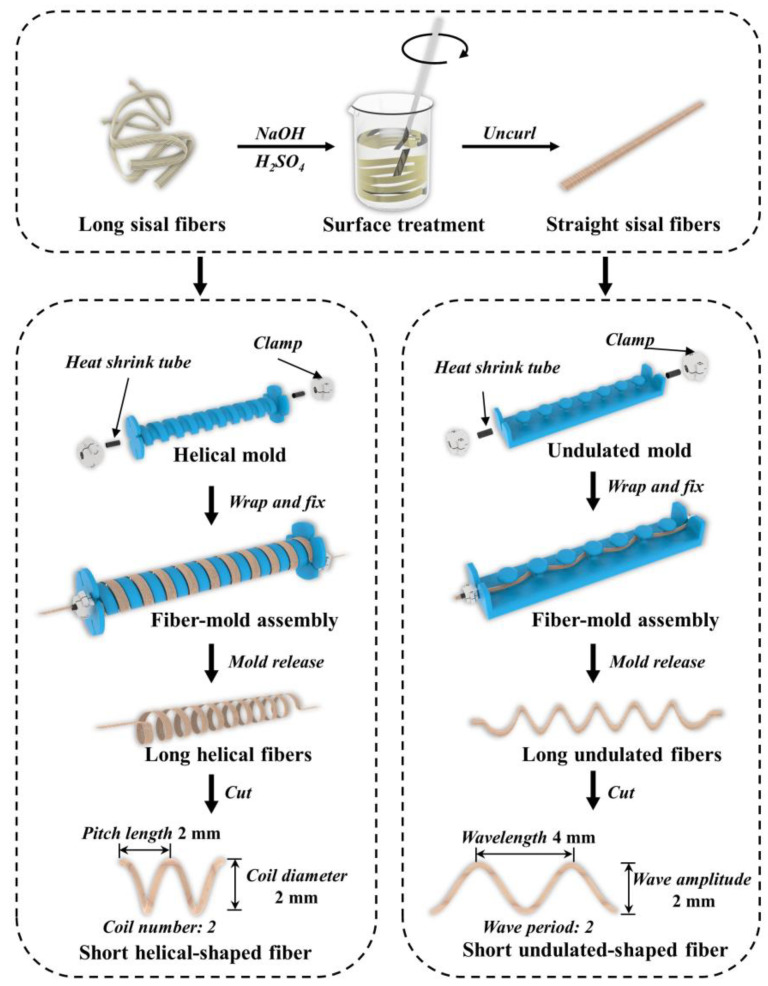
Preparation process of sisal fibers with different shapes.

Sisal fibers (*Agave sisalana*) were purchased from Dongfang Sisal Group Co. (Zhanjiang, China). In this study, the sisal fibers were made into helical, undulated, and straight shapes; the corresponding preparation process is shown schematically in Figure 1. The long sisal fibers (40 cm in length and 0.5 mm in diameter) were firstly water-washed and naturally air-dried for several days. The dried fibers were then subjected to the following surface treatment: dipped in 2 wt.% NaOH solution for 90 min, steeped in 1 wt.% H_2_SO_4_ solution for 30 min, and rinsed with distilled water until the solution was neutral. After this, to obtain the different fiber shapes, the sisal fibers were wrapped and fixed to laboratory-made molds, including a helical mold and an undulated mold, and the ends of the fibers were fastened with clamps and heat shrink tubes. The fiber–mold assemblies were then placed in a drying oven and heat-treated at 70 °C until the fiber shape was fully formed. Finally, the three types of sisal fibers were cut into 10–13 mm lengths. The detailed parameters of the helical-shaped and undulated-shaped fibers are given in Figure 1.

### 2.2. Fabrication of the Composite Materials

The formulation of the developed friction composite materials is given in Table 1. Among the listed ingredients, sisal fibers and compound mineral fibers were used as reinforcement components (25 wt.%); phenolic resin was used as a binder component (14 wt.%); petroleum coke, graphite, zinc stearate, antimony sulfide, porous iron powder, and alumina were used as friction modifier components (35 wt.%); and barium sulfate, friction powder, and vermiculite powder were used as filler components (26 wt.%). According to the shapes of the sisal fibers, the prepared composite materials were designated as FMSF, FMUF, and FMHF, respectively, corresponding with the straight-shaped, undulated-shaped, and helical-shaped fibers.

**Figure 2:**
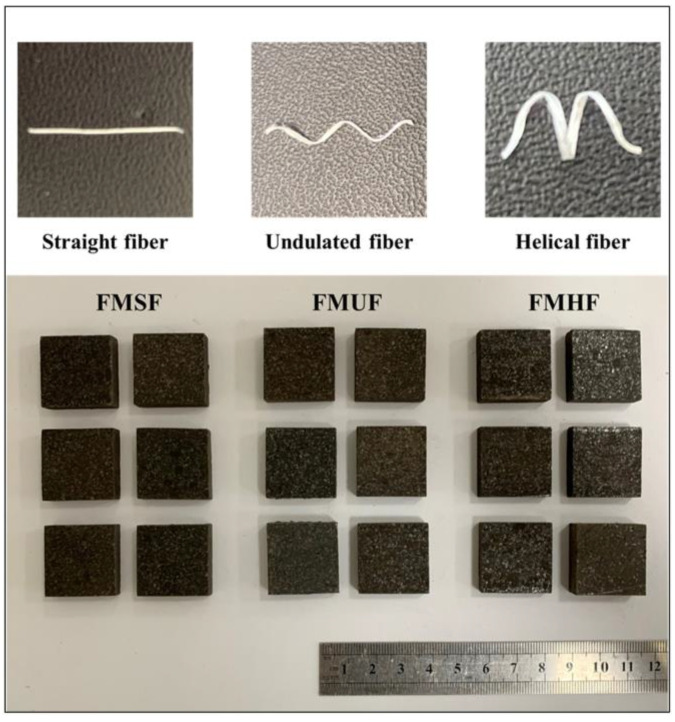
The friction composite samples after fabrication.

The friction composite materials were fabricated using a compression molding technique, which consisted of the following four steps: mixing, hot pressing, post-curing, and finishing. The detailed conditions of the fabrication process of the composite materials are described in Table 2. The obtained friction composite samples after fabrication are shown in Figure 2.

### 2.3. Characterization of the Developed Composite Materials

The developed composite materials were subjected to characterization, including their physical, mechanical, and tribological as well as morphological properties. All tests were repeated at least three times to ensure data accuracy and reliability.

#### 2.3.1. Density Test

The density (*ρ*) of FMSF, FMUF, and FMHF was measured by an electronic balance (MP-5002, Wuxi, China) according to the Archimedes drainage method and was calculated as follows [33]:

(1) $$\rho =\frac{m_{1}}{m_{1}-m_{2}}\times \rho_{w}$$

where *m*_1_ is the dry weight of the tested composites in air (g), *m*_2_ is the submerged weight of the composites in distilled water (g), *ρ*_w_ is the density of distilled water (g/cm^3^; here, *ρ*_w_ = 1 g/cm^3^).

#### 2.3.2. Hardness Test

The hardness of FMSF, FMUF, and FMHF was detected using a Rockwell hardness apparatus (HRSS-150, Chongqing, China) at the R scale according to the Chinese National Standard (CNS) GB/T5766-2007. In the hardness test, the initial load and main load were set as 98.07 N and 490.3 N, respectively, and the diameter of the steel ball indenter was 12.7 mm.

#### 2.3.3. Impact Property Test

The impact strength of FMSF, FMUF, and FMHF was determined by an impact testing instrument (XJ-40A, Shanghai, China) as per the CNS GB/T 5764-2011. After the test, the fracture surfaces of these composite materials were inspected using SEM (EVO-18, Zeiss, Jena, Germany) at a 20 kV voltage.

#### 2.3.4. Evaluation of the Tribological Behaviors 

**Figure 3:**
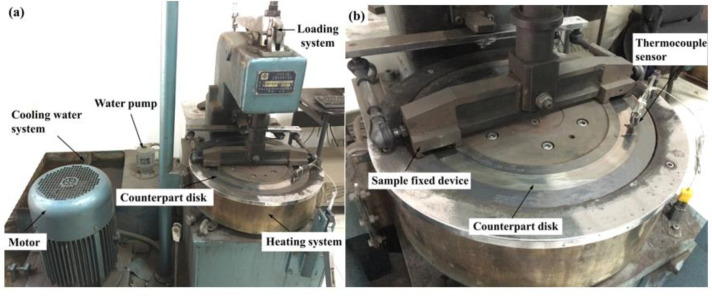
Experimental set-up: (**a**) constant-speed friction testing machine and (**b**) sample fixed device.

The tribological characteristics of FMSF, FMUF, and FMHF were assessed using a constant-speed friction testing apparatus (JF150D-II, Changchun, China), shown schematically in Figure 3. The tribological tests were conducted based on the CNS GB/T5763-2008 and comprised a fade part and a recovery part. The detailed test conditions used for this study are given in Table 3. During testing, a rotating disk was driven by an electric motor, the normal pressure was regulated using a loading system, and the friction force between the composite materials and the disk was monitored using a tension–compression sensor. The testing temperatures were detected by a thermocouple sensor and remained at the preset values using an electric heating system and a cooling water system. The friction coefficient (*μ*) of the tested polymer composites was automatically stored. The thickness loss and weight loss of the composite materials for each test were measured by a spiral micrometer and an electronic balance (0.0001 g accuracy), respectively, and then the corresponding wear rate (*W*) was determined using Formula (2) [34].

(2) $$W=\frac{1}{2\pi R}\times \frac{A}{N}\times \frac{\Delta h}{f}$$

where *R* is the measuring radius of the counterpart disk (mm); *A* is the area of the polymer composite (mm^2^); *N* is the rotating number during the test; Δ*h* is the thickness loss of the polymer composite (mm); *f* is the average friction force (N) (here, *A* = 625 mm^2^ and *R* = 150 mm, respectively).

The friction coefficients of the polymer composites decrease temporarily at elevated temperatures and should be regained at lower temperatures, which are referred to as fade and recovery, respectively [35]. These characteristics are of critical importance in the performance evaluation of friction composite materials. The fade rate and recovery rate (*F* and *R*) were evaluated based on Formulas (3) and (4), respectively [25].

(3) $$F=\frac{\mu_{F100{}^{\circ}C}-\mu_{F350{}^{\circ}C}}{\mu_{F100{}^{\circ}C}}\times 100\%$$

(4) $$R=\frac{\mu_{R100{}^{\circ}C}}{\mu_{F100{}^{\circ}C}}\times 100\%$$

where *μ*_F100°C_ and *μ*_R100°C_ are the *μ* at a temperature of 100 °C during the fade test and recovery test, respectively; *μ*_F350°C_ is the *μ* at a temperature of 350 °C during the fade test.

#### 2.3.5. Morphological Characterization

The worn surface features of FMSF, FMUF, and FMHF were inspected using SEM at a 20 kV voltage to obtain the corresponding wear mechanisms. The composite materials for the SEM observations were gold-sputtered prior to inspection to make these tested composite materials conductive to the conditions. The three-dimensional profiles and surface roughness of FMSF, FMUF, and FMHF were measured using confocal laser scanning microscopy (CLSM, OLS3000, OLYMPUS, Beijing, China).

## 3. Results and Discussion

### 3.1. Physical and Mechanical Performance Analysis

The test results of the physical and mechanical performance for the developed friction composite materials are given in Table 4. The composites FMSF, FMUF, and FMHF exhibited roughly similar density values, almost all around 2.21 g/cm^3^, indicating that the sisal fiber shapes exerted no obvious effect on the density of the polymer composite systems. Similarly, the hardness values of FMSF, FMUF, and FMHF also did not differ significantly, and only changed in a narrow range of 93.4–94.1 HRR. In general, the addition of natural fibers can reduce both the density and hardness of fiber-reinforced polymer composites [23,36], but in this study, the content of sisal fiber added in each group was the same so the overall density and hardness values of the composites FMHF and FMUF were relatively close to those of the composite FMSF.

**Figure 4:**
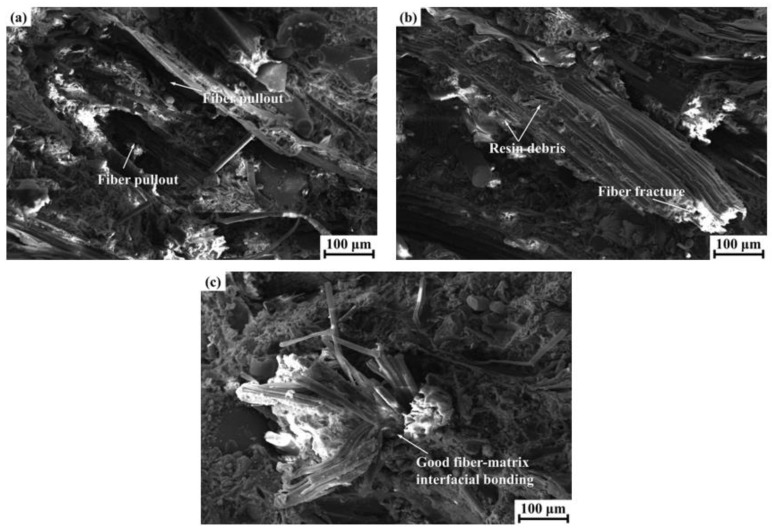
Impact fracture surface morphologies of the tested composite materials: (**a**) FMSF; (**b**) FMUF; (**c**) FMHF.

An impact strength test was conducted to assess the impact resistance property of the polymer composites and to estimate the brittleness and toughness to a certain extent. As is apparent from Table 4, the impact strength values followed the order FMHF > FMUF > FMSF, which indicates that both the helical and the undulated fibers had positive effects on the impact property of the composite materials. Generally, the impact behaviors of fiber-reinforced friction materials are directly related to the interfacial conditions between the reinforcing fibers and the composite matrix [37]. To more accurately explain the above test results, the impact fracture surfaces of these composite materials were examined and are presented in Figure 4. For the composite FMSF (Figure 4a), we observed that the sisal fibers were almost completely pulled out of the polymer composite, leaving obvious cavities on the fractured surface that provided evidence for the poor interface adhesion between the straight fibers and the resin matrix. In the case of the composite FMUF (Figure 4b), the fiber fracture and pullout appeared on the fracture surface; a small amount of resin debris remained on the pulled fibers, resulting from the enhanced fiber–matrix interface bonding. For the composite FMHF (Figure 4c), the sisal fibers were tightly integrated with the composite matrix and presented a fatigue fracture under the applied load, showing the preferable interface condition between the helical sisal fibers and the composite matrix. These observations were consistent with the impact strength results.

### 3.2. Tribological Behavior Analysis

#### 3.2.1. Friction Behavior

**Figure 5:**
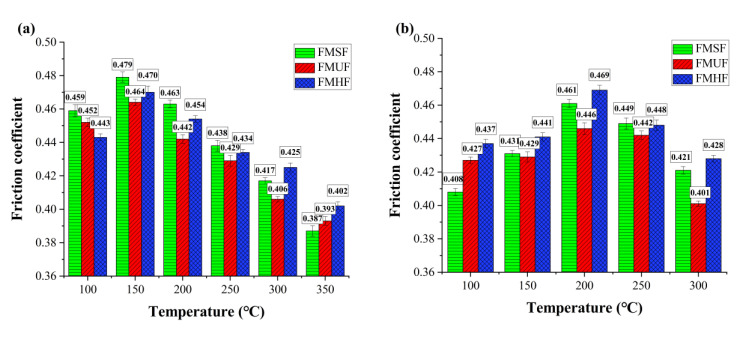
Friction performance of the developed composite materials in (**a**) fade testing and (**b**) recovery testing.

A friction test was performed to assess the influence of the fiber shape on the friction performance of these composite materials. The results of *μ* for the composites FMSF, FMUF, and FMHF at different testing temperatures are displayed in Figure 5. As is apparent from Figure 5a, in the fade testing, the overall change trend of *μ* for each friction composite was similar; that is, it increased at first and then decreased with an increase in the testing temperature from 100 to 350 °C. Such behavior was due to the decrease in the shear strength and the thermal degradation of the organic components such as the phenolic resin and the sisal fiber as well as the compound mineral fiber at elevated testing temperatures, which was consistent with the previous reports of Ma et al. [38] and Cai et al. [39]. It should be noted that the friction values of the tested composite materials fluctuated within the range of 0.387–0.479, which was in accordance with the CNS GB/T5763-2008.

In the recovery testing, as illustrated in Figure 5b, the *μ* of the composites FMSF, FMUF, and FMHF initially increased at testing temperatures between 300 and 200 °C, and then decreased as the temperature changed from 200 to 100 °C. The general reason for this behavior was the generation of wear debris and the rheological behaviors between the wear debris and the friction surface layer [1,40]. Overall, the *μ* values of all tested polymer composites presented a relatively stable range of 0.401–0.469, which could positively affect the braking stability of the friction composite systems.

The fade rate and the recovery rate are two important parameters in a friction performance evaluation and reflect the specific changes in *μ* during fade and recovery tests. The results of the fade and recovery rates of the developed composite materials are given in Table 5. The fade rate for the present work was as follows: FMSF > FMUF > FMHF. The recovery rate showed the following order: FMHF > FMUF > FMSF. This indicates that the composite FMHF with helical-shaped fibers exhibited improved frictional stability, followed by the composite FMUF with undulated-shaped fibers. It is understood that the fade and recovery behaviors of fiber-reinforced composite materials are mainly related to the stability of the friction interface, especially the improved fiber–matrix bonding strength and the generated secondary plateaus [6,41,42]. A detailed discussion about the friction surface topography of the tested composite materials is given in Section 3.3.

#### 3.2.2. Wear Behavior

**Figure 6:**
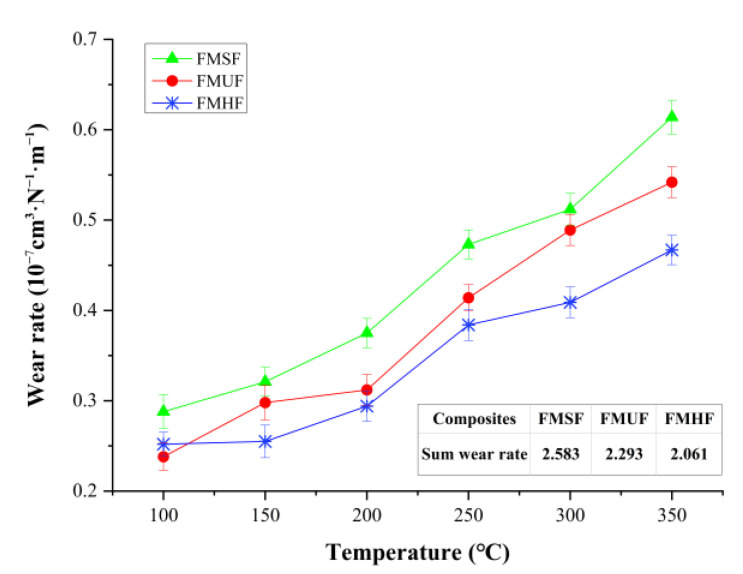
Wear performance of the developed composite materials.

Wear resistance is a significant performance indicator that directly affects the service life of friction composite systems. In this paper, a wear performance test was conducted to assess the effect of the sisal fiber shape on the wear resistance of the developed composite materials. The corresponding test results are presented in Figure 6, which clearly shows that the overall trend of the wear rate for the composites FMSF, FMUF, and FMHF was similar and it exhibited an obvious increasing trend as the testing temperature increased. This behavior was caused by the softening and thermal degradation of the binder components with the increasing temperature, which then deteriorated the adhesion conditions at the filler–matrix interface and even resulted in the debonding and separation of the filler–matrix, thereby leading to increased wear rates. Similar results were observed by Manoharan et al. [43] and Wang et al. [44]. It could also be seen that the composites FMHF and FMUF provided lower wear rates in comparison with the composite FMSF at high temperatures, which may be associated with the enhanced interfacial bonding strength between the sisal fibers and the composite matrix [45]. The sum wear rate of each tested polymer composite was calculated (Figure 6) and it followed the trend of FMSF > FMUF > FMHF, indicating that the helical fibers led to a marked improvement in the wear behavior of the composite materials, followed by the undulated fibers. The detailed reasons for the above observations are given in the section below.

### 3.3. Worn Surface Analysis

**Figure 7:**
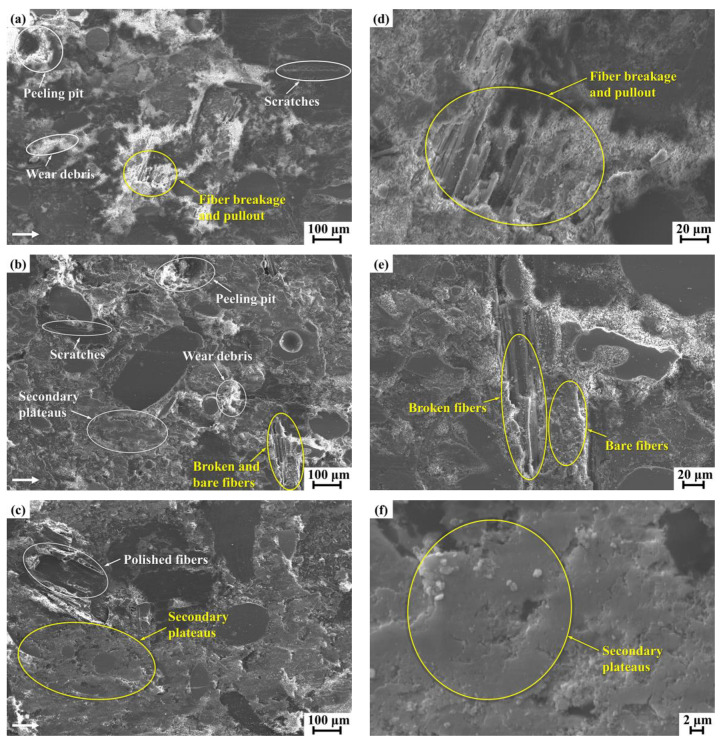
The worn surface morphologies of the tested composite materials: (**a**,**d**) FMSF; (**b**,**e**) FMUF; (**c**,**f**) FMHF.

SEM observations were conducted to examine the worn surface features of the tested composite materials, which helped to ascertain the corresponding wear mechanisms and interpret the differences in the tribological properties. The worn surface morphologies of the composites FMSF, FMUF, and FMHF after the completion of the tribological test are given in Figure 7. In the case of the composite FMSF (Figure 7a), several peeling pits and wear scratches along the sliding direction appeared on its surface; a large amount of wear debris and particles and a few secondary plateaus were also discovered. The fibers suffered from severe debonding, and pullout and slide-off under the applied friction force even occurred due to the poor interface adhesion condition (Figure 7d), which could be responsible for the poor tribological performance of this composite. During the friction process, wear particles and pieces were generated, and most of these particles played an abrasive role, ploughing and abrading the friction surfaces and causing three-body abrasion wear in the friction composite materials [46]. The presence of peeling pits was probably due to the detachment of unstable materials under the action of shear forces, showing the adhesive wear characteristics [36]. From the above surface features, it could be inferred that abrasive wear and adhesive wear were the dominant wear mechanisms for the composite FMSF.

For the composite FMUF (Figure 7b), the worn surface showed evidence of a reduced number of wear pieces and particles, wear scratches, and peeling pits, together with a few secondary plateaus. Interestingly, the fibers were partially adhered in the composite matrix and the rest presented fiber breakage and pullout due to the limited fiber–matrix interface bonding quality (Figure 7e), which could support moderate tribological behavior. In general, the existence of secondary plateaus played an essential role in determining the friction and wear characteristics of the friction composite materials. During friction, the primary plateaus were produced by the reinforcement fibers and thermally stable components, which acted as barriers to prevent wear debris movement. The entrapped wear debris then accumulated and compacted at the friction interface under frictional pressure and heat, resulting in the generation of the secondary plateaus. Such behaviors have been reported by Kumar et al. [47] and Kchaou et al. [48], in which secondary plateaus were a significant contributing factor for promoting friction stability and wear resistance in resin-based friction materials. The composite FMHF, as displayed in Figure 7c, exhibited a relatively good-quality worn surface as there was strong bonding at the fiber–matrix interface. The extent of fiber debonding and pullout was much less than in the composites FMUF and FMSF. Many stable secondary plateaus adhered to the polymer matrix (Figure 7f) and a reduced amount of wear scratches and fine wear particles appeared on the surface, which helped to support the best friction and wear performance of this composite.

**Figure 8:**
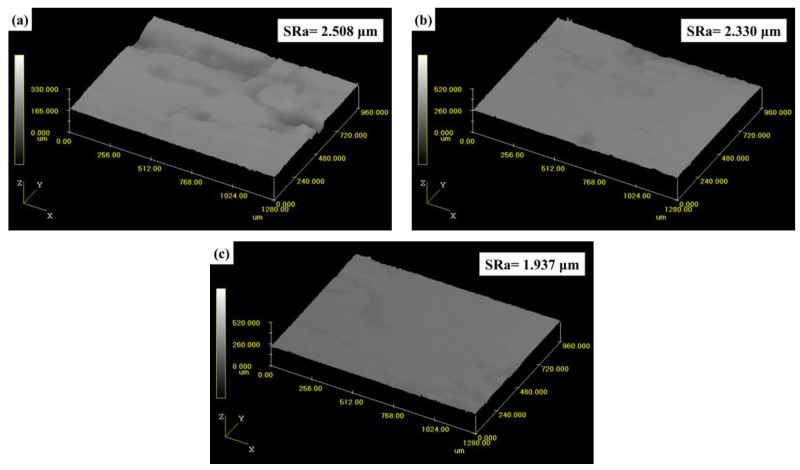
The three-dimensional profiles and surface roughness of the tested composite materials: (**a**) FMSF; (**b**) FMUF; (**c**) FMHF.

CLSM observations were carried out to accurately measure the worn surface roughness of the tested composite materials, which was associated with the friction and wear properties to a certain extent. The results of the surface roughness (SRa) of FMSF, FMUF, and FMHF, and the corresponding three-dimensional reconstructions of the surface geometry of these composites, are presented in Figure 8. As is apparent from Figure 8, the SRa values followed the order FMSF > FMUF > FMHF, indicating that the composite FMSF (SRa = 2.508 μm) and composite FMHF (SRa = 1.937 μm) exhibited the highest and lowest roughness, respectively. Generally, under the dry sliding condition, the higher surface roughness was mainly due to the serious damage of the friction surface of the polymer composites, which directly showed an increase in SRa values [37]. The surface roughness results were consistent with the tribological behaviors and morphological analysis results.

**Figure 9:**
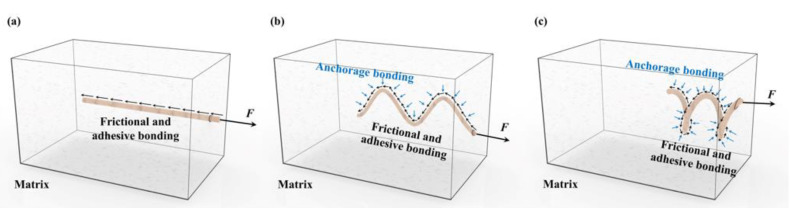
Anchorage bonding and frictional bonding between the fibers and the composite matrix: (**a**) straight fibers; (**b**) undulated fibers; (**c**) helical fibers.

From the above observations, the helical sisal fibers in the polymer composite provided superior interface bonding between the fibers and the resin matrix compared with the undulated and straight sisal fibers. This may be ascribed to the fact that the helical-shaped fibers exhibited not only frictional and adhesive bonding at the fiber–matrix interfaces but also anchorage bonding resulting from the mechanical contributions of the fiber deformations based on their three-dimensional characteristics, thus leading to a higher resistance to fiber debonding and pullout during the friction process than the other two types of fibers (Figure 9) [49,50]. Thus, it can be concluded that the sisal fibers with the helical shape are a superior candidate as a reinforcement material in resin-based friction composites.

## 4. Conclusions

In this paper, resin-based friction composite materials with different shapes of sisal fibers were fabricated and evaluated from the perspective of their tribological, mechanical, physical, and morphological performance. The following conclusions could be obtained:

1. The shapes of the sisal fibers exerted no obvious influence on the density and hardness of the composite materials, whereas they showed a noticeable effect on the impact strength. The composite FMHF exhibited the highest impact strength (0.584 J/cm^2^).
2. From a friction performance point of view, the composite FMHF provided enhanced fade resistance and recovery characteristics and it showed a relatively low fade rate (9.26%) and a high recovery rate (98.65%). This was followed by the composite FMUF and the composite FMSF.
3. From a wear behavior point of view, the wear resistance of the composite FMHF was found to be superior when compared with the composites FMUF and FMSF and it exhibited the sum wear rate of 2.061 × 10^–7^ cm^3^∙N^−1^∙m^−1^.
4. An SEM analysis demonstrated that adding the helical-shaped sisal fibers could enhance the fiber–matrix interface bonding quality, reduce the extent of fiber debonding and pullout, and provide more secondary plateaus adhered to the friction surface. These were responsible for the overall behavior changes in the composite FMHF. Thus, the above results clearly prove that helical-shaped sisal fibers are a more suitable candidate for resin-based friction composite material applications.

## Figures and Tables

**Table 1:** Formulation of the prepared composite materials.

Classification	Ingredients	Content (wt.%)
Reinforcements	Sisal fiber	6
	Compound mineral fiber	19
Binders	Phenolic resin	14
Friction modifiers	Petroleum coke	5
	Graphite	8
	Zinc stearate	2
	Antimony sulfide	3
	Porous iron powder	10
	Alumina	7
Fillers	Barium sulfate	19
	Friction powder	2
	Vermiculite powder	5
	Total	100

**Table 2:** Detailed conditions involved in the fabrication process of the friction materials.

S. No.	Step	Conditions
1	Mixing (JF805R paddle-type blender)	Total duration 10 min, mixing sequence of ingredients: reinforcements (2 min), followed by fillers and friction modifiers (5 min) and finally binders (3 min).
2	Hot pressing (JFY50 thermocompressor)	Pressure 40 MPa, temperature 165 °C, curing time 30 min with three intermittent breathings.
3	Post-curing (JF980B heat-treatment machine)	Temperatures of 140, 160, 180 °C for 1, 3, 6 h, respectively.
4	Finishing	Cut into 25 mm × 25 mm × 6 mm size.

**Table 3:** Tribological test conditions details.

Conditions	Fade Test	Recovery Test
Temperature (°C)	100, 150, 200, 250, 300, 350	300, 250, 200, 150, 100
Rotating speed (rpm)	480	480
Load (MPa)	0.98	0.98
Rotating number	5000	7500
Counterpart disk	HT250 cast iron disk (HB 180–220)	
Test parameters	Friction coefficient, fade rate, recovery rate, wear rate	

**Table 4:** Physical and mechanical properties of the tested composite materials.

Properties	FMSF	FMUF	FMHF
Density (g/cm^3^)	2.21	2.21	2.20
Hardness (HRR)	93.4	94.1	93.8
Impact strength (J/cm^2^)	0.496	0.533	0.584

**Table 5:** Fade and recovery rates of the developed composite materials.

Properties	FMSF	FMUF	FMHF
Fade rate	15.69%	13.05%	9.26%
Recovery rate	88.89%	94.47%	98.65%

## Data Availability

Not applicable.
